# Invasive Plant *Alternanthera philoxeroides* Benefits More Competition Advantage from Rhizosphere Bacteria Regardless of the Host Source

**DOI:** 10.3390/plants12112085

**Published:** 2023-05-24

**Authors:** Xu Li, Yi Zhang, Fang-Li Kong, Misbah Naz, Jian-Yu Zhou, Shan-Shan Qi, Zhi-Cong Dai, Dao-Lin Du

**Affiliations:** 1 School of Emergency Management, Jiangsu University, Zhenjiang 212013, China; 2 Institute of Environment and Ecology, School of the Environmental and Safety Engineering, Jiangsu University, Zhenjiang 212013, China; 3 Key Laboratory of Modern Agricultural Equipment and Technology, Ministry of Education, School of Agricultural Engineering, Jiangsu University, Zhenjiang 212013, China; 4 Jiangsu Collaborative Innovation Center of Technology and Material of Water Treatment, Suzhou University of Science and Technology, Suzhou 215009, China

**Keywords:** invasive plant, competitive ability, invasion, rhizosphere microorganisms, sterile seedlings

## Abstract

The rhizosphere plays a vital role in the exchange of materials in the soil–plant ecosystem, and rhizosphere microorganisms are crucial for plant growth and development. In this study, we isolated two strains of *Pantoea* rhizosphere bacteria separately from invasive *Alternanthera philoxeroides* and native *A. sessilis*. We conducted a control experiment to test the effects of these bacteria on the growth and competition of the two plant species using sterile seedlings. Our findings showed that the rhizobacteria strain isolated from *A. sessilis* significantly promoted the growth of invasive *A. philoxeroides* in monoculture compared to native *A. sessilis*. Both strains significantly enhanced the growth and competitiveness of invasive *A. philoxeroides* under competition conditions, regardless of their host source. Our study suggests that rhizosphere bacteria, including those from different host sources, can contribute to the invasion of *A. philoxeroides* by significantly enhancing its competitiveness.

## 1. Introduction

The rhizosphere, the zone of soil around plant roots, serves as a complex interface where soil, microorganisms, and host plants interact with each other [1]. Plants use the root secretion of amino acids, carbohydrates, and other compounds to selectively attract specific microorganisms which, in turn, receive carbon sources and nutrients for their growth. This process involves complex interactions between the plant and the microorganisms in the rhizosphere [2]. The microorganisms present in the rhizosphere are crucial for various aspects of plant development, including nutrient uptake, growth, resistance to environmental stressors, and in the prevention of diseases [3,4,5].

In recent years, the rapid growth of economies and international trade has led to a significant increase in the severity of biological invasions, making it a critical environmental problem on a global scale [6] These invasions are now recognized as an essential contributor to global change [7]. In addition, biological invasions have emerged as the second greatest threat to biological diversity [8]. Invasive species can reduce the biodiversity of native species, modify the ecological direction of native species, and affect the structure of communities through competition, ecological niche contest, predation, transmission of parasitic/pathogenic organisms, and so on [9]. Currently, numerous non-native plant species have resulted in significant ecological impacts in various regions around the globe [10,11]. The global ecological impacts caused by the introduction of non-native plant species, commonly referred to as invasive species, to new regions, have been significant. These plants can outcompete native species, disrupt ecosystem processes, and even drive native species to extinction [12]. Efforts are underway to prevent the introduction of new invasive species and to control the spread of those that have already become established. Preventing the introduction and spread of invasive plant species is an important part of conservation efforts [13].

Invasive plants have the ability to change the structure and diversity of plant communities in the areas they invade [14,15] but can also influence the structure and function of soil microbial communities [16]. Soil microbes play an important role in the establishment of invasive plants and may also be a driving factor in plant invasion [17]. Previous studies have shown that invasive plants can recruit different soil microorganisms to promote their growth [18]. Invasive plants can alter soil biomes and thus promote plant invasion [19]. For example, *Bromus tectorum* can alter and disturb the composition and structure of rhizosphere mycorrhizal communities [20,21,22]. The invasive species *Prosopis juliflora* can alter its root bacterial and fungal community diversity, thereby enhancing its root colonization and increasing dry biomass and plant phosphorus, and supporting its growth and invasion [23,24]. *Alnus trabeculosa* increases soil bacterial diversity in invaded areas [25]. Furthermore, studies have demonstrated that invasive plants employ various strategies to evade the inhibitory effects of soil pathogens [26,27]. The presence of certain microbes in the rhizosphere of *Mikania micrantha,* which are involved in nutrient acquisition and pathogen suppression, significantly enhances the plant’s ability to adapt and invade various environments [28]. Invasive plants may influence soil nutrient content through the soil microbial community [29,30]. For example, the invasive tree *Staghorn sumac* changed the structure of the soil community of nitrogen-fixing bacteria to increase soil N utilization efficiency [31]. The invasion of *Flaveria bidentis* altered the community structure of *Bacillus*, whose recruitment promotes the growth of *F. bidentis* by increasing the levels of nitrogen and phosphorus in the plant [29]. The invasive plant *Ambrosia artemisiifolia* increased the availability of soil nitrogen and phosphorus by recruiting certain *Bacillus* species, thereby enhancing its competitive growth and facilitating its successful invasion [32]. 

There are many studies on the interaction between single strains of bacteria and invasive plants, but all of them involve genera such as AMF, *Bacillus*, *Pseudomonas*, *Rhizobium*, and *Pseudarthrobacter* sp. For example, Wang et al. [33] isolated two strains of nitrogen-fixing bacteria, *Pseudarthrobacter* sp. and *Ensifer* sp., from the rhizosphere of *Solidago canadensis*, which were able to alleviate nutrient stress and promote the root development of *S. canadensis* in low-nitrogen environments. Qi et al. [34] found that *G. intraradices* could aid the acquisition of insoluble phosphorus by *S. canadensis*, reducing the plant’s resource investment in the belowground part and enhancing the investment in the aboveground part. Although *Pantoea* has also been isolated from the rhizosphere and endosphere of invasive plants, there are few studies on the relationship between *Pantoea* and invasive plants. The genus *Pantoea*, isolated from a variety of sources, contains a number of versatile species. It has been reported that it has been isolated from the rhizosphere and endosphere of various plants such as potato, rice, cucumber, and citrus, as well as from the intestines of some pests. Studies have shown that *Pantoea* can promote host plant growth and development through phosphorolysis, the stimulation of phytohormone production, and the induction of plant systemic resistance [35]. For example, Suman et al. [36] isolated *Pantoea agglomerans* and *Pantoea ananatis* from maize rhizospheres, both of which have the ability to dissolve phosphorus and produce iron carriers and IAA, and an inoculation with these two strains significantly promoted the growth of maize, rice, and wheat. In addition, *Pantoea* can be used as a biocontrol agent to suppress pathogenic bacteria. Ahmet Akk¨oprü et al. [37] found that inoculation with *P. agglomerans*, an endophytic bacterium isolated from cucumber leaves, was effective not only in reducing the severity of angular leaf spot disease (ALS), but also in increasing the yield of cucumber. Bi et al. [38] isolated a strain of *Pantoea vagans* strain BWL1 from the surface of citrus and found that it could show its resistance to *Penicillium expansum* by producing metabolites to inhibit the biosynthesis of ergosterol.

Native to South America, *Alternanthera philoxeroides* (Mart.) Griseb., which is also known as “alligator weed”, is an invasive alien plant that is widely distributed across the globe. It is a herbaceous perennial weed that can grow in both terrestrial and aquatic environments, having a strong phenotypic plasticity as well as a fast reproduction rate, forming dense populations and thus causing loss of biodiversity [39,40]. Since its introduction to China in the 1930s, its range has expanded rapidly northwards, where it can reproduce by shoots and stems; it is now mainly distributed between 21 °N and 36.8 °N [41]. It is found in the Yangtze River basin and southern provinces such as Guangdong, Guizhou, Yunnan, and Fujian, and has had an enormous influence on the ecosystem as well as social economy of China [42]. Its native congener, *Alternanthera sessilis*, is a native Chinese annual or perennial herb that can be propagated by seeds, stems, and shoots. The latitudinal range of *A. sessilis* overlaps exactly with that of *A. philoxeroides* in mainland China [40]. Both species can form dense communities on land and often occur simultaneously in natural habitats in China [39]. This study aimed to address the research questions by investigating the potential of rhizosphere bacteria from *Alternanthera* to promote the growth of *A. philoxeroides* and *A. sessilis*, and to determine which of these plant species could benefit more from the presence of rhizosphere bacteria in competition. We isolated two strains of bacteria from the rhizosphere of *A. philoxeroides* and *A. sessilis.* Greenhouse experiments with microbial inoculation were also conducted to test the following hypothesis: both strains of rhizosphere bacteria will promote the growth of *A. philoxeroides* under monoculture conditions and improve its competitiveness.

## 2. Results

### 2.1. Identification of Strains

**Figure 1:**
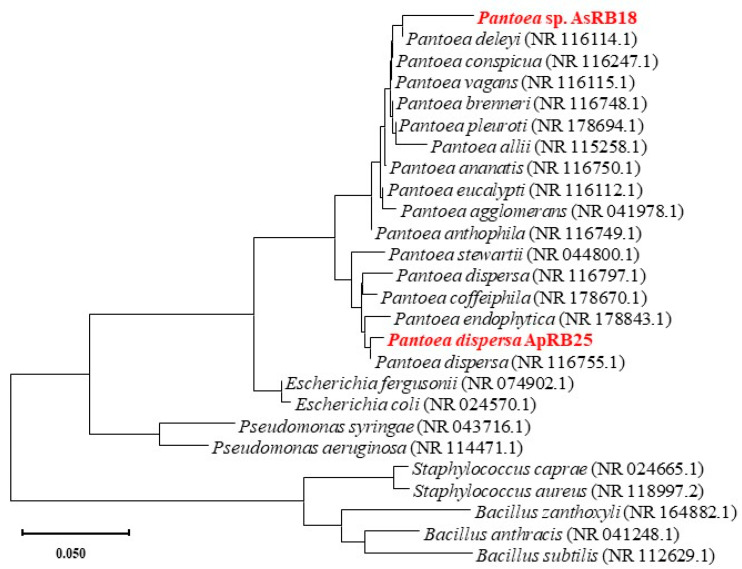
Phylogenetic tree of the rhizobacteria strain AsRB18 isolated from *A. sessilis* and strain ApRB25 isolated from *A. philoxeroides*.

Two rhizosphere strains were isolated and phylogenetic trees were constructed to identified their species (Figure 1); we isolated one strain in the rhizosphere of *A. philoxeroides*, named as *Pantoea dispersa* ApRB25 (Ap—*A. philoxeroides*, RB—rhizosphere bacteria), and another strain, which belongs to the same genus in the rhizosphere of *A. sessilis*, named as *Pantoea* sp. AsRB18 (As—*A. sessilis*, RB-rhizosphere bacteria).

### 2.2. Effect of Rhizosphere Bacteria on the Growth of Invasive A. philoxeroides and Native A. sessilis

The clonal growth and biomass of *A. philoxeroides* were significantly affected by different planting patterns or rhizosphere bacteria inoculation. Furthermore, the interaction between these two factors had a significant effect on the node number of *A. philoxeroides*. However, except for the significant effect of different planting patterns on the clonal growth and biomass of *A. sessilis*, there was no significant effect of rhizosphere bacterial inoculation and its interaction with planting patterns on the clonal growth and biomass of *A. sessilis* (Table 1).

**Figure 2:**
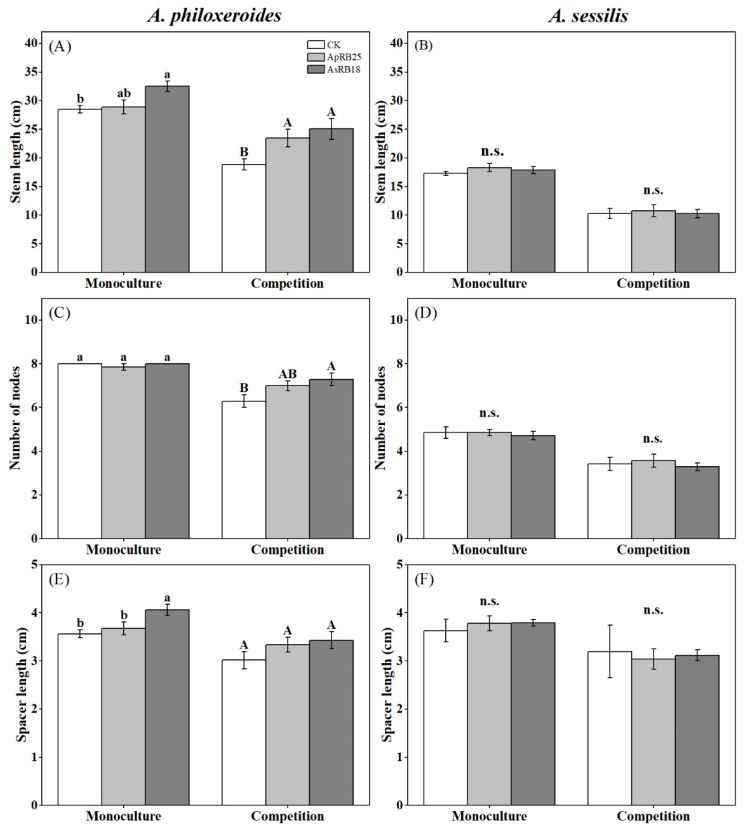
The effects of rhizosphere bacteria and plantation patterns on stem length (**A**,**B**), number of nodes (**C**,**D**), and spacer length (**E**,**F**) of *A. philoxeroides* and *A. sessilis* (CK, no rhizosphere bacteria inoculated, only sterile 0.9% NaCl solution was added). Error bars are the S.E. (*n* = 7). Different letters indicate a significant difference at *p* < 0.05. n.s. means no significant difference.

The inoculation of strain ApRB25 from *A. philoxeroides* rhizosphere did not significantly affect the growth of *A. philoxeroides* in the monoculture (Figure 2A,C,E). Inoculating the *A. sessilis* rhizosphere strain AsRB18 had a significant impact on promoting the spacer length of *A. philoxeroides*, as shown in (Figure 2E). Meanwhile, in competitive conditions, inoculating the *A. philoxeroides* rhizosphere strain ApRB25 significantly increased the stem length of *A. philoxeroides* (Figure 2A). In addition, the inoculation of strain AsRB18 from *A. sessilis* rhizosphere had a different effect on *A. philoxeroides*; the stem length and node number of *A. philoxeroides* were also significantly promoted (Figure 2A,C). However, neither the ApRB25 or the AsRB18 strain had significant effects on the clonal growth of *A. sessilis* under monoculture or competition conditions (Figure 2B,D,F).

**Figure 3:**
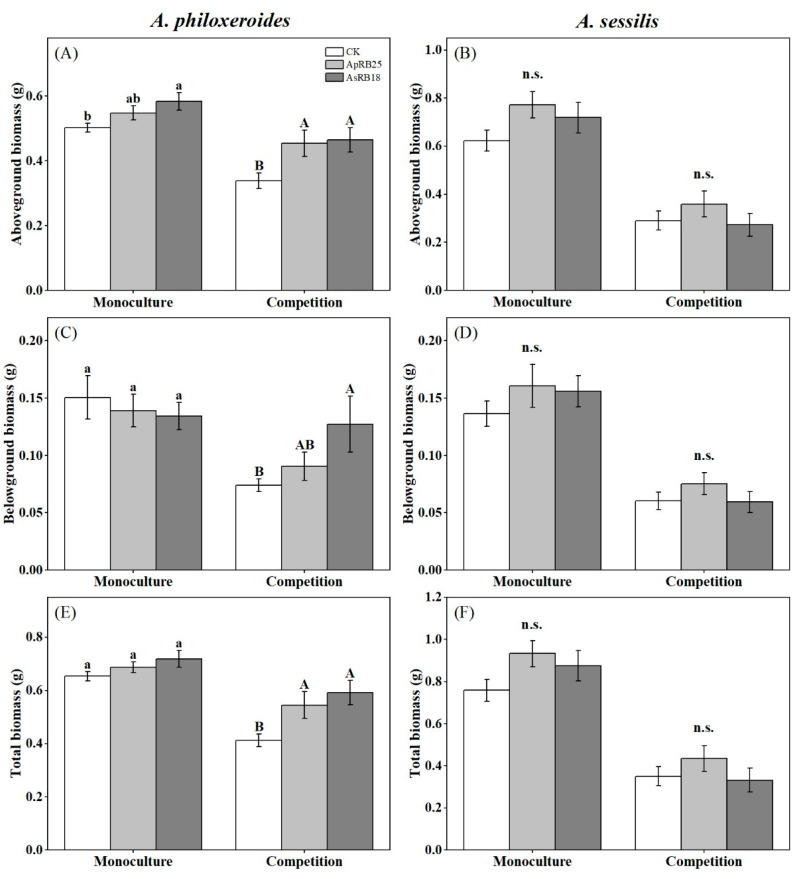
The effects of rhizosphere bacteria and plantation patterns on aboveground biomass (**A**,**B**), belowground biomass (**C**,**D**), and total biomass (**E**,**F**) of *A. philoxeroides* and *A. sessilis* (CK, no rhizosphere bacteria inoculated, only sterile 0.9% NaCl solution was added). Error bars are the S.E. (*n* = 7). Different letters indicate a significant difference at *p* < 0.05. n.s. means no significant difference.

With the inoculation of the *A. sessilis* rhizosphere strain AsRB18, the aboveground biomass of *A. philoxeroides* was significantly increased in monoculture (Figure 3A). In competition, the aboveground, belowground, and total biomass of *A. philoxeroides* were significantly promoted by the inoculation of strain AsRB18. The inoculation of the *A. philoxeroides* rhizosphere strain ApRB25 also promoted its own biomass and total biomass in the competition treatment. However, there was no significant change in biomass (Figure 3B,D,F) for native *A. sessilis* under monoculture or competition conditions after the inoculation of strains ApRB25 or AsRB18.

### 2.3. Effect of Rhizosphere Bacteria on the Relative Competitive Intensity Index (RCI) of A. philoxeroides and A. sessilis

**Figure 4:**
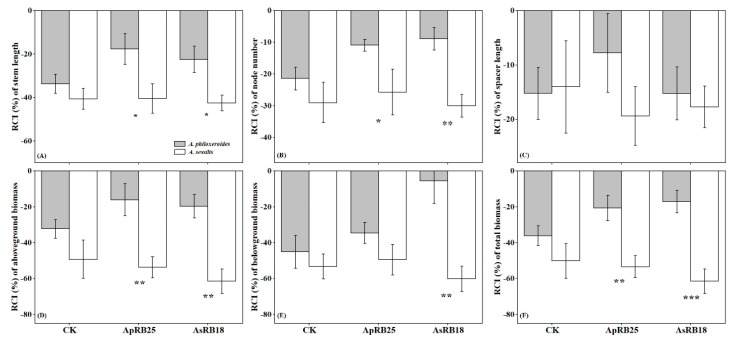
The effect of rhizosphere bacteria on relative competitive intensity index (RCI) of *A. philoxeroides* and *A. sessilis* (CK, no rhizosphere bacteria inoculated, only sterile 0.9% NaCl solution was added). (**A**) RCI of stem length, (**B**) RCI of node number, (**C**) RCI of spacer length, (**D**) RCI of aboveground biomass, (**E**) RCI of belowground biomass, and (**F**) RCI of total biomass. Error bars are the S.E. (*n* = 7). * means *p* < 0.05, ** means *p* < 0.01, and *** means *p* < 0.001.

The RCI values for the clonal growth and biomass of *A. philoxeroides* and *A. sessilis* were found to be less than 0, indicating that interspecific competition hindered the growth of both plant species. No significant difference was observed in the relative competitive intensity index (RCI) values for the clonal growth and biomass in the absence of rhizosphere bacteria inoculation (CK treatment) (Figure 4). When inoculated with strains ApRB25 and AsRB18, the RCI values for the stem length and node number were significantly higher for *A. philoxeroides* than for *A. sessilis* (Figure 4A,B). Additionally, the RCI values for the aboveground biomass and total biomass of *A. philoxeroides* were significantly higher than those of *A. sessilis* after inoculation with strain ApRB25 (Figure 4D,F). The RCI values for the aboveground, belowground, and total biomass of *A. philoxeroides* were also significantly higher than those of *A. sessilis* after inoculation with strain AsRB18 (Figure 4D–F).

## 3. Discussion

We conducted a study to examine the impact of rhizosphere bacteria belonging to the same genus, but from different host sources, on the growth and competition of the invasive plant *A. philoxeroides* and the native plant *A. sessilis*. Our findings indicate that, regardless of the host source, rhizosphere bacteria had a significant promotional effect on the clonal growth and competition ability of invasive *A. philoxeroides,* but had no effect on the native *A. sessilis.*

Plant-associated microbes significantly affect plant performance and play crucial roles in the successful invasion of alien species [43]. Rhizosphere microorganisms have a positive effect on plant growth, nutrient uptake, and disease suppression [4]. Previous studies showed that invasive plants can recruit different soil microbes to enhance their own growth [19]. In this study, we found that the bacteria from native plant rhizospheres could promote the invasive plant growth and competition. This might contribute to the invasion of the clonal plant *A. philoxeroides*. How, then, might the rhizosphere bacteria work on it? Clonal growth provides a plant with the ability to produce new plants that share resources such as minerals, carbohydrates, and water [44], facilitating the growth and development of meristems or the production of new meristems, and facilitating access to resources for the clonal plants [45]. Clonal organs (stolons and rhizomes) can act as sites for storing carbohydrates or soluble proteins, enhancing plant survival and reproduction, which may be a way for plants to cope with environmental disturbances [44,46,47,48,49]. As a successful invasive and clonal plant, *A. philoxeroides* might be subject to environmental disturbances in new habitats during invasive colonization [41,49]. Inoculation with the rhizosphere bacteria significantly increased the clonal growth of *A. philoxeroides* (Figure 2), especially under competitive conditions. This differs from the first hypothesis, where the rhizosphere bacteria strain isolated from *A. sessilis* significantly promoted the growth of invasive *A. philoxeroides* in monoculture compared to native *A. sessilis*. The better clonal growth enhanced by rhizosphere bacteria might promote their ability to occupy space in new habitats [46] and thus predict the spatial structural pattern of their growth and reproduction [50]. Therefore, *A. philoxeroides* achieves population expansion through the clonal reproduction at invaded sites [39,51,52]; this may enhance its ability to expand populations. 

Invasive species tend to have a competitive advantage over native species, and many invasive plants can significantly impact the community structure and ecological function of rhizosphere microorganisms [53]. The recruitment of different soil microbes by invasive plants to alter the soil microbial community near their roots is a potential mechanism for successful invasive plants to influence nutrient cycling [17,54,55,56]. Studies have found that microorganisms contribute to the invasion of invasive plants [33,34,45,57]. Microorganisms promote the growth and development of host plants through nitrogen fixation, indoleacetic acid production, and iron carrier production. RCI [58] values are used to indicate the competitive ability of a species, with higher RCI values indicating the greater competitive ability of the species. In the present study, the RCI of *A. philoxeroides* was significantly higher than that of native *A. sessilis* after inoculation with rhizosphere bacteria isolated from *A. philoxeroides* and *A. sessilis* (Figure 4). This is consistent with the second hypothesis. The findings indicate that the promotion of rhizosphere bacteria could be a contributing factor in the higher competitive abilities of the invasive *A. philoxeroides* compared to the native *A. sessilis*. Additionally, this promotion of microorganisms in new habitats could facilitate the successful invasion of *A. philoxeroides* and support the symbiosis hypothesis [59]. Further research is necessary in order to investigate the internal mechanisms by which rhizosphere bacteria promote the competitive ability of invasive plants.

Differences in environment and host plants lead to geographic differences in soil microbial community structure and function. Studies have shown that the invasion of alien plants is one of the reasons for the existence of geographic differences [27]. For example, Ferrari et al. [60] isolated a strain of *Rhizobia* from the invasive Argentine plant *Robinia pseudoacacia* that was more efficient in N_2_-fixing than native N_2_-fixing bacteria. Since the same population of plants and plant counterparts of rhizosphere bacteria were used in this study, the final results obtained may be specific. Therefore, the role of microorganisms in plant invasion can be verified in the future by collecting samples from several invasive sites of *A. philoxeroides* and analyzing their soil microbial community structure and function.

## 4. Materials and Methods

### 4.1. Sample Collection

Both the whole plants of *A. philoxeroides* and *A. sessilis* were collected in September 2020 from Fuzhou Forest Park in Fujian Province (26°14′24.43″ N, 119°29′30.57″ E). The complete root systems were excavated with shovels and, together with the rhizosphere soil, were sealed in plastic bags and kept at 4 °C. After about 48 h, the rhizosphere soil was used for the isolation and identification of rhizosphere bacteria. Both of the two species’ stems were propagated in a greenhouse at Jiangsu University, Zhenjiang, China (119°31.76′ E, 32°12.02′ N).

### 4.2. Sterile Seedlings

To avoid the effects of other microorganisms, sterile seedlings were used to assess the roles of rhizosphere bacteria in plant growth. Both *A. philoxeroides* and native *A. sessilis* sterile seedlings were derived from their shoots, and the sterile seedling system was established by referencing Dai et al. [61]. Firstly, plant shoots were washed with 75% ethanol for 1 min and soaked in 10% NaClO for 10 min. Then, the shoots were washed five times with sterile water. Secondly, the basal ends of these shoots were slowly inserted into sterilized Murashige and Skoog (MS) solid medium and cultured in sterile culture flasks. After 60 days of growth, the stem segments with two stem nodes were cut and set aside for future experiments.

### 4.3. Isolation and Phylogenetic Analysis of Bacterial Strains

The method for isolating rhizosphere bacteria strains was modified from that of Ofek-Lalzar [62] and Sarah Croes [63] as follows: (1) soil shaken from root was collected and grounded well, then 5 g of the soil sample was weighed in a 150 mL triangular flask with 45 mL of sterile PBS and glass beads using an autoclaved spoon and shaken for 30 min (200 rpm, 30 °C); (2) the suspension was transferred to a new 250 mL triangular flask in a ultra clean bench, 50 mL of sterile PBS was added, this was shaken continuously for 30 min and repeated 2–3 times to collect the suspension for use; (3) 1 mL of the suspension was taken and diluted with sterile PBS at concentrations of 10^−5^, 10^−6^, and 10^−7^, and 300 μL of each concentration was applied onto LB liquid medium, with three replicates per concentration; (4) the coated dishes were placed in a constant-temperature incubator (30 °C) and incubated for 5 d, protected from light, until no more new colonies grew; (5) a single clone was placed in a tube containing sterilized LB liquid medium on an ultra-clean table and incubated in a shaker overnight for 24 h (200 rpm, 30 °C); (6) 800 μL of bacterial solution was taken in a sterilized glycerol tube (containing 200 μL glycerol), labeled and sealed, then the glycerol tubes were stored in an ultra-low-temperature refrigerator at −80 °C. 

### 4.4. 16S rRNA Identification and Construction of Phylogenetic Tree

Bacteria were collected by centrifugation (10,000 rpm, 5 min) from the solute, kept in centrifuge tubes, and re-suspended by adding 200 μL of sterile water as a PCR template for the solute to be used. The target fragment was amplified by PCR using 16S-rRNA universal primers 27F (5′-AGAGTTTGATCCTGGCTCA-3′) and 518R (5′-ATTACCGCGGCTGCTGG-3′). The PCR amplification conditions were: pre-denaturation at 95 °C for 5 min; 95 °C (30 s), 52 °C (30 s), 72 °C (30 s), 30 cycles; and extension at 72 °C for 10 min. After amplification, the PCR products were detected by electrophoresis using a 1% agarose gel, and then sent to Shanghai Biotechnology Service Co. (Sangon Biotech (Shanghai) Co., Ltd. (China)) for sequence determination. The resulting sequences were analyzed by comparison using the online BLAST tool (http://blast.ncbi.nlm.nih.gov/Blast.cgi, (accessed on 10 November 2020)), submitted to the GenBank database with accession number OQ654038 and OQ654039, and then imported into MEGA_X_10.1.7 followed by using the Neighbor joining method [64], the Kimura2-parametric model (Kimura2-parametermodel) [65], and 1000 iterations (Bootstrap method) using the self-sampling method [66] to construct the phylogenetic tree.

### 4.5. Common Garden Experiment

To assess the effects of rhizosphere bacteria from different host sources on the growth and competition of *A. philoxeroides* and *A. sessilis,* a microbial inoculation experiment was conducted in April 2022 in a greenhouse at Jiangsu University. The rhizosphere bacteria *Pantoea dissersa* ApRB25, isolated from *A. philoxeroides*, and the rhizosphere bacteria *Pantoea* sp. AsRB18, isolated from native *A. sessilis*, were used for further experiments. These two strains were inoculated in LB liquid medium and incubated for 24 h at 30°C with shaking (200 rpm). Bacterial cells were collected by centrifugation, washed three times with sterile 0.9% NaCl, and then prepared into an OD_600_ = 1.0 suspension with sterile 0.9% NaCl solution for the inoculation. Washed river sand sterilized at 121 °C for 2 h was used as the substrate for pot culture in plastic paper cups (8.6 × 7 × 5.3 cm). 

In monoculture, we grew one sterile seedling of *A. philoxeroides* or *A. sessilis* in media as above with three different bacteria inoculation treatments, which is: control treatment (CK, no rhizosphere bacteria inoculated, only sterile 0.9% NaCl solution was added), inoculated with the strain ApRB25 (hereafter referred to as “ApRB25”, 2 mL suspension of strain ApRB25 with OD_600_ = 1.0 was added), and inoculated with the strain AsRB18 (hereafter referred to as “AsRB18”, 2 mL suspension of strain AsRB18 with OD_600_ = 1.0 was added). To quantify the roles of rhizosphere bacteria in competition, one sterile seedling of *A. philoxeroides* and one sterile seedling of *A. sessilis* were planted in pots with three bacteria inoculation treatments as above. That is, there were 6 treatments (3 inoculation treatments × 2 planting patterns) with 7 replicates for each treatment. An amount of 0.5 × Hoagland nutrient solution was added to all the pots every week to provide nutrition. Sterile purified water was added to all the plants when needed. All the pots were put into the greenhouse with natural light and at 28 °C. 

### 4.6. Growth Trait Measurements

After 70 days of growth, all plants were harvested. Shoot length, node number, and spacer length was measured. Roots of each seedling were carefully removed from the media. Aboveground and belowground parts were separated and dried at 60 °C for 48 h to collect biomass data. Total biomass was calculated with the sum of aboveground and belowground biomass. The relative competitive intensity index (RCI) [58] was calculated to quantify the effect of rhizosphere bacteria on the competitiveness of two plant species. The RCI was calculated according to:

RCI (%) = (X − Y)/Y(1)

In Equation (1), X represents the total biomass of invasive *A. philoxeroides* or native *A. sessilis* under competition, and Y represents the total biomass of *A. philoxeroides* or native *A. sessilis* under monoculture.

### 4.7. Data Analysis

Data were processed using SPSS 25.0 software. One-way ANOVA and “Duncan’s test” (*p* < 0.05) were used to analyze the effects of different strains on the growth of *A. philoxeroides* and *A. sessilis*. One-way ANOVA and Student’s t test (*p* < 0.05) were used to analyze the effect of different strains on the RCI of *A. philoxeroides* and native *A. sessilis*, and two-way ANOVAS (*p* < 0.05) were used to analyze the effect of different strain treatments and different planting patterns on plant growth. 

## 5. Conclusions

We found that the rhizosphere bacteria strain isolated from native *A. sessilis* significantly promoted the growth of invasive *A. philoxeroides* in monoculture. Both strains isolated from native and invasive *Alternanthera* remarkably enhanced the growth and competitiveness of invasive *A. philoxeroides* under competitive conditions, regardless of their host origin. It was shown that rhizobacteria from different host sources can promote the invasion of *A. philoxeroides* by enhancing its competitiveness. 

## Figures and Tables

**Table 1:** The growth of *A. philoxeroides* and *A. sessilis* was analyzed through two-way ANOVAs, taking into account the different strain and planting pattern treatments.

Source of Sample	Factor	df	*A. philoxeroides*		*A. sessilis*	
			*F*	*p*	*F*	*p*
Stem length	Strains	2	8.391	0.001	0.508	0.606
	Plantations	1	53.813	<0.001	142.283	<0.001
	Strains × Plantations	2	1.462	0.245	0.098	0.907
Number of nodes	Strains	2	3.265	0.05	0.429	0.655
	Plantations	1	46.676	<0.001	51.49	<0.001
	Strains × Plantations	2	3.794	0.032	0.061	0.941
Spacer length	Strains	2	4.94	0.013	0.016	0.984
	Plantations	1	18.137	<0.001	7.884	0.008
	Strains × Plantations	2	0.552	0.581	0.189	0.828
Aboveground biomass	Strains	2	7.002	0.003	2.348	0.11
	Plantations	1	28.004	<0.001	90.365	<0.001
	Strains × Plantations	2	0.754	0.478	0.652	0.527
Belowground biomass	Strains	2	0.818	0.449	1.317	0.281
	Plantations	1	11.897	0.001	75.341	<0.001
	Strains × Plantations	2	2.487	0.097	0.36	0.7
Total biomass	Strains	2	6.723	0.003	2.483	0.098
	Plantations	1	37.36	<0.001	102.187	<0.001
	Strains × Plantations	2	1.669	0.203	0.682	0.512

## Data Availability

The sequence data that support the findings of this study have been deposited into GenBank of National Center for Biotechnology Information (https://www.ncbi.nlm/nih.gov/genbank/, (accessed on 10 November 2020)) with accession number OQ654038 and OQ654039. The data presented in this study are available on request from the corresponding author (e-mail: daizhicong@163.com).
